# A comparative study of dose distribution of PBT, 3D-CRT and IMRT for pediatric brain tumors

**DOI:** 10.1186/s13014-017-0775-2

**Published:** 2017-02-22

**Authors:** Daichi Takizawa, Masashi Mizumoto, Tetsuya Yamamoto, Yoshiko Oshiro, Hiroko Fukushima, Takashi Fukushima, Toshiyuki Terunuma, Toshiyuki Okumura, Koji Tsuboi, Hideyuki Sakurai

**Affiliations:** 1 Department of Radiation Oncology, University of Tsukuba, Tsukuba, Ibaraki Japan; 2 Department of Neurosurgery, University of Tsukuba, Tsukuba, Ibaraki Japan; 3 Department of Child Health, University of Tsukuba, Tsukuba, Ibaraki Japan; 4 Proton Medical Research Center, Graduate School of Comprehensive Human Sciences, University of Tsukuba, Tsukuba, Ibaraki Japan

**Keywords:** Brain, 3D-CRT, IMRT, PBT, Pediatric tumor

## Abstract

**Introduction:**

It was reported that proton beam therapy (PBT) reduced the normal brain dose compared with X-ray therapy for pediatric brain tumors. We considered whether there was not the condition that PBT was more disadvantageous than intensity modulated photon radiotherapy (IMRT) and 3D conventional radiotherapy (3D-CRT) for treatment of pediatric brain tumors about the dose reduction for the normal brain when the tumor location or tumor size were different.

**Methods:**

The subjects were 12 patients treated with PBT at our institute, including 6 cases of ependymoma treated by local irradiation and 6 cases of germinoma treated by irradiation of all four cerebral ventricles. IMRT and 3D-CRT treatment plans were made for these 12 cases, with optimization using the same planning conditions as those for PBT. Model cases were also compared using sphere targets with different diameters or locations in the brain, and the normal brain doses with PBT, IMRT and 3D-CRT were compared using the same planning conditions.

**Results:**

PBT significantly reduced the average dose to normal brain tissue compared to 3D-CRT and IMRT in all cases. There was no difference between 3D-CRT and IMRT. The average normal brain doses for PBT, 3D-CRT, and IMRT were 5.1–34.8% (median 14.9%), 11.0–48.5% (23.8%), and 11.5–53.1% (23.5%), respectively, in ependymoma cases; and 42.3–61.2% (48.9%), 54.5–74.0% (62.8%), and 56.3–72.1% (61.2%), respectively, in germinoma cases. In the model cases, PBT significantly reduced the average normal brain dose for larger tumors and for tumors located at the periphery of the brain.

**Conclusion:**

PBT reduces the average dose to normal brain tissue, compared with 3D-CRT and IMRT. The effect is higher for a tumor that is larger or located laterally.

## Introduction

Deterioration of intelligence after radiotherapy is an important problem in growing children. The degree of deterioration is affected by the irradiation dose, volume, site, and age at irradiation [1]. A major concern of brain irradiation in pediatric patients is subsequent deterioration of intelligence [6–10], since neurodevelopment is affected by the treatment dose and age at irradiation [1, 11, 12]. In 4 patients among 27 children with medulloblastoma treated with craniospinal irradiation with a posterior fossa boost, Walter et al. [8] found an IQ decline of 3.9 points per year during a median observation period of 4.8 years, and cognitive losses did not seem to have reached a plateau. Merchant et al. analysed the correlation with the degree of deterioration and DVH of the whole brain and suggested that IQ can be obtained from the following formula [1]:

$$ \mathrm{I}\mathrm{Q} = 93.11 \pm \left(0.028 \times \mathrm{age}\ \hbox{-}\ 0.0095 \times \mathrm{average}\ \mathrm{dose}\ \mathrm{to}\ \mathrm{the}\ \mathrm{brain}\right) \times \mathrm{time} $$

Intensity modulated photon radiotherapy (IMRT) is now widely used and can reduce the dose to an at-risk organ and decrease the high dose area, but the low dose area is wider than 3D-CRT. [13] X. Sharon et al. found that IMRT redused temporal lobe dose compared with 3D-CRT [14], but it is possible that the dose reduction for temporal lobe by IMRT increase the dose of the other lobes, and don’t reduce average dose to the brain compare with 3D-CRT. In contrast, proton beam therapy (PBT) has a sharp energy peak, referred to as the Bragg peak, which produces excellent dose localization and reduces the dose to normal tissue [2, 15, 16]. Therefore, excellent tumor coverage can be achieved with a small number of beam ports, and several reports have shown advantages of PBT compared to photon radiotherapy, including IMRT. [3] The advantages of PBT compared to photon radiotherapy including IMRT have been described in treatment of pediatric CNS tumors [9–11]. MacDonald et al. found that proton beams can achieve tumor coverage as well as IMRT, but that normal tissue sparing was better in PBT for patients with germ cell tumor and ependymoma [17, 18].

We conduct PBT for all pediatric patients with brain malignancies who are indicated for photon radiotherapy in order to reduce the normal brain dose [4, 5]. However, the conditions under which the advantages of PBT are maximized are unclear; for example, a large or small tumor, local or whole-ventricle irradiation, and a peripheral or central tumor. In this study, we evaluated the more advantageous condition of PBT for pediatric brain tumors in comparison to 3D-CRT and IMRT, using quantitative analysis of localized irradiation for ependymoma cases and whole-ventricular irradiation for germinoma cases, and model cases that targets were different size or location.

## Patients and methods

### Patients

The subjects were 12 pediatric patients (Table 1) with brain tumors treated with PBT at our institute from 2009 to 2011. The study was approved by the institutional review board at our institution. Six patients (3 males, 3 females; median age 4 [range: 2–6] years old) had ependymoma and 6 had germinoma (3 males, 3 females; median age 13 [range: 10–16] years old). Localized and whole-ventricle irradiation was performed for ependymoma and germinoma, respectively, during which the patients were immobilized using individually manufactured thermoplastic masks.

**Table 1:** Clinical background of patients

No.	Age (y)	Sex	Tumor	CTV1 (cc)	Dose to normal brain (%) [3D-CRT]	Dose to normal brain (%) [IMRT]	Dose to normal brain (%) [PBT]	The relative decrease (%) (3DCRT–PBT)/3DCRT	The relative decrease (%) (IMRT–PBT)/IMRT
1	6	M	Ependymoma	6.9	11.0	11.5	5.1	53.6	55.7
2	6	M	Ependymoma	74.0	20.5	20.1	14.4	29.8	28.4
3	2	F	Ependymoma	6.1	17.8	17.1	6.5	63.5	62.0
4	3	F	Ependymoma	42.5	30.0	31.8	15.4	48.7	51.6
5	4	M	Ependymoma	116.3	48.5	53.1	34.8	28.2	34.5
6	3	F	Ependymoma	48.0	27.0	26.9	16.7	38.1	37.9
7	11	M	Germinoma	344.8	74.0	72.1	61.2	17.3	15.1
8	16	F	Germinoma	150.2	60.5	61.8	48.5	19.8	21.5
9	10	M	Germinoma	168.8	54.5	56.3	42.3	22.4	24.9
10	14	M	Germinoma	200.6	63.0	62.7	49.2	21.9	21.5
11	14	F	Germinoma	216.7	67.0	60.6	55.0	17.9	9.2
12	12	F	Germinoma	353.8	62.5	58.9	47.4	24.2	19.5

### Comparison of PBT, IMRT, and 3D-CRT

Proton beams from 155 to 250 MeV, generated through a linear accelerator and synchrotron, were spread out and shaped with ridge filters, double-scattering sheets, multicollimators, and custom-made boluses to ensure that the beams conformed to the treatment planning data. Planning CT images were taken at 2-mm intervals. IMRT and 3D-CRT treatment plans were generated and optimized using the same practical treatment planning CT as that used for PBT to compare dose distributions among the three methods. The prescribed doses were the same in each cases among the three methods, 45Gy to 61.2Gy (median 52.2Gy) in ependymoma cases as local irradiation, and 24Gy to 30.6Gy (median 30.6Gy) in germinoma cases as whole ventricle irradiation. All PBT plans were prescribed the same dose with IMRT/3DCRT as the equivalent dose. The clinical target volume (CTV) for ependymoma was defined as the surgical defect plus a margin of 0.5 to 1 cm. The CTV for germinoma was defined as all cerebral ventricles. The same planning target volume (PTV) and at-risk organ was re-contoured for the photon radiotherapy plans. The PTV was identical for PBT, IMRT, and 3D-CRT for each patient and was defined by the 95% iso-dose line in all plans. The maximal dose prescriptions for at-risk organs were <4 Gy for the lens and <50 Gy for the brainstem and chiasma. We used helical tomotherapy that delivered 51 of discrete gantry positions per rotation for IMRT planning, and two or more gantry angles that made the minimum average normal brain dose and didn’t pass the eyes for 3D-CRT and PBT planning. SOBP for PBT plans were prescribed by a 1 cm unit and chosed the smallest width as far as PTV covers were enough in the condition. Leaf margin of the PBT plans and 3D-CRT plans also optimized the smallest width as far as PTV covers were enough in the condition. All planning optimization were checked two radiotherapists. Statistical analysis was performed using t-test in SPSS (SPSS Inc., Chicago, IL, USA). Dose-volume histograms (DVHs) for the normal brain were calculated for PBT, IMRT, and 3D-CRT and compared among the methods.

### Model cases

To assess the advantages of PBT based on tumor size and location, model target spheres of different sizes and locations were evaluated. Targets were first prepared with the iso-center at the center of the brain and diameters of 2, 3, 4, and 6 cm to analyze the effect of target size. Secondly, a target of 4 cm in diameter was moved from the center of the brain to the peripheral region horizontally in a right-left direction (x-axis) at 1-cm intervals to analyze the effect of target location. Dose distributions for each target were calculated for PBT, 3D-CRT, and IMRT, and the normal brain dose was evaluated as a percentage of the prescription dose. The relative decrease in normal brain dose in PBT compared to 3DCRT or IMRT was calculated from the equation:

$$ \mathrm{The}\ \mathrm{relative}\ \mathrm{decrease}\ \mathrm{in}\ \mathrm{brain}\ \mathrm{average}\ \mathrm{dose}=\frac{\mathrm{IMRT}\ \mathrm{or}\ 3\mathrm{D} - \mathrm{CRT}\ \mathrm{brain}\ \mathrm{average}\ \mathrm{dose} - \mathrm{PBT}\ \mathrm{brain}\ \mathrm{average}\ \mathrm{dose}}{\ \mathrm{IMRT}\ \mathrm{or}\ 3\mathrm{D}-\mathrm{CRT}\ \mathrm{brain}\ \mathrm{average}\ \mathrm{dose}} $$

## Results

### Ependymoma cases (local irradiation)

**Fig. 1:**
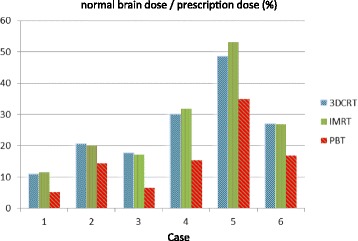
Average normal brain doses for patients with ependymoma

Doses to normal brain tissue in PBT, 3D-CRT, and IMRT for the 6 patients with ependymoma are shown in Fig. 1. In the respective methods, the average normal brain doses were 15.5, 25.8, and 26.8% of the prescription dose. Normal brain doses were significantly lower in PBT compared to 3D-CRT (*p* = 0.001) and IMRT (*p* = 0.003), with differences ranging from 5.7 to 18.3% (median 10.5%). The relative decreases were 28.2 to 63.4% (median 43.4%) compared to 3D-CRT and 28.4 to 62.0% (median 44.7%) compared to IMRT. There was no significant difference in the normal brain dose between IMRT and 3D-CRT (*p* = 0.296).

### Germinoma cases (whole-ventricle irradiation)

**Fig. 2:**
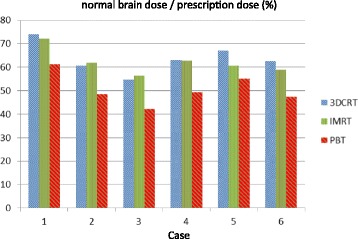
Average normal brain doses for patients with germinoma

Doses to normal brain tissue in PBT, 3D-CRT, and IMRT for the 6 patients with germinoma are shown in Fig. 2. The average normal brain doses were 50.6, 63.6, and 62.1% of the prescription dose, respectively. Normal brain doses were significantly smaller in PBT compared to 3D-CRT and IMRT (both *p* = 0.000), with differences ranging from 5.6 to 15.1% (median 12.5%). The relative decreases were 17.3 to 24.1% (median 20.9%) compared to 3D-CRT and 9.2 to 24.9% (median 20.5%) compared to IMRT. There was no significant difference in the normal brain dose between IMRT and 3D-CRT (*p* = 0.287).

**Fig. 3:**
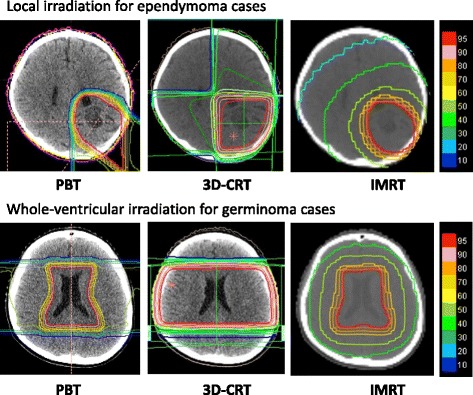
The example of the treatment planning of ependymoma and germinoma in each methods

Figure 3 shows the example of the treatment planning of ependymoma and germinoma in each methods.

### Model cases

**Fig. 4:**
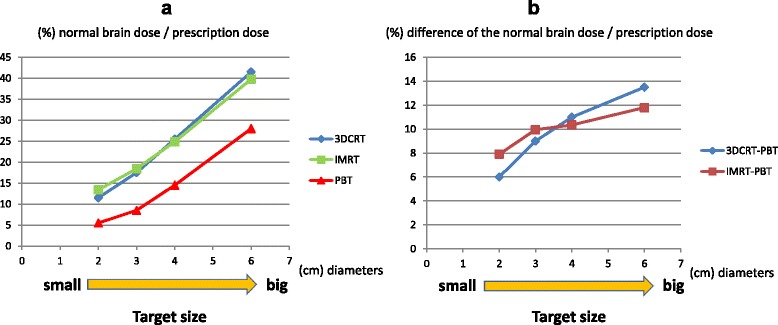
**a** Average normal brain dose for each treatment plan with sphere targets with the same iso-center as the brain center and different diameters (2, 3, 4, 6 cm). **b** Correlation between target size and difference in normal brain dose in PBT compared to 3DCRT or IMRT

The normal brain doses for targets of diameters 2, 3, 4, and 6 cm with the iso-center at the brain center were 5.5, 8.5, 14.5, and 28% of the prescription dose in PBT; 11.5, 17.5, 25.5, and 41.5% in 3D-CRT; and 13.4, 18.5, 24.9, and 39.8% in IMRT, respectively (Fig. 4). For all targets, the average normal brain dose was smaller in PBT plans compared to 3D-CRT (*p* = 0.008) and IMRT (*p* = 0.001), with no significant difference between 3D-CRT and IMRT (*p* = 0.886). There were significant positive correlations between target size and normal brain dose (*p* = 0.000 for PBT, *p* = 0.002 for 3D-CRT, *p* = 0.003 for IMRT); if the target was larger, the average normal brain dose was larger in all three methods. There were also significant positive correlations between target size and the difference in normal brain dose in PBT compared to 3D-CRT (*p* = 0.023) and IMRT (*p* = 0.048); if the target was larger, there was a larger reduction in normal brain dose in PBT compared to radiotherapy.

**Fig. 5:**
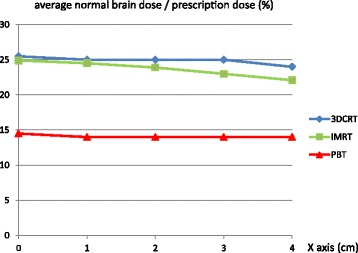
Average normal brain dose for each treatment plan, using bilateral irradiation for 3D-CRT and PBT, with sphere targets with the same diameter and different iso-centers at 0, 1, 2, 3, and 4 cm from the brain center

Regarding target location, and with use of bilateral irradiation for 3D-CRT and PBT, the average normal brain doses for targets with iso-centers at 0, 1, 2, 3, and 4 cm from the brain center were 14.5%, 14.0%, 14.0%, 14.0%, 14.0% of the prescription dose for PBT; 25.5, 25.0, 25.0, 25.0, and 24.0% for 3D-CRT; and 24.9, 24.5, 23.9, 23.0% and 22.1% for IMRT, respectively (Fig. 5). For all target locations, the normal brain doses were significantly smaller in PBT plans compared to 3D-CRT and IMRT (both *p* = 0.000), and in IMRT plans compared to 3D-CRT (*p* = 0.018). There was no correlation between target location and normal brain dose in PBT (*p* = 0.182). In 3D-CRT, the normal brain dose tended to decrease when the target was more peripheral, but the difference was not significant (*p* = 0.058). In IMRT, the normal brain dose was significantly decreased when the target moved peripherally (*p* = 0.016). The difference in normal brain dose between PBT and 3D-CRT was not significantly correlated with target location (*p* = 0.182), but that between PBT and IMRT was significantly correlated with target location (*p* = 0.014).

**Fig. 6:**
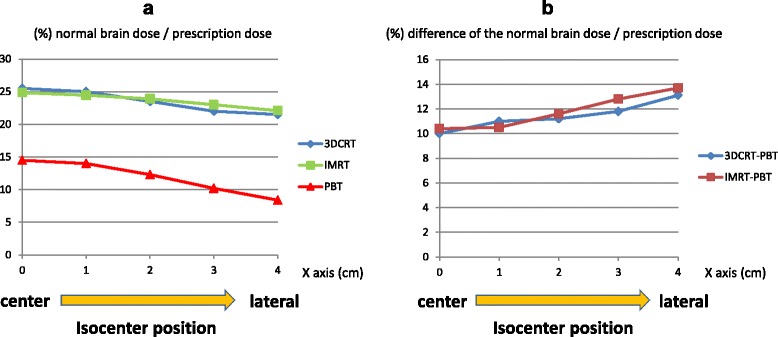
**a** Average normal brain dose for each treatment plan, using two oblique angle irradiation for 3D-CRT and PBT, with sphere targets with the same diameter and different iso-centers at 0, 1, 2, 3, and 4 cm from the brain center. **b** Correlation between target position and difference in normal brain dose in PBT compared to 3DCRT or IMRT

Using two oblique angle irradiation for 3D-CRT and PBT, the average normal brain doses for targets with iso-centers 0, 1, 2, 3, and 4 cm from the brain center were 14.5%, 14.0%, 12.3%, 10.2%, 8.4% of the prescription dose for PBT; and 25.5, 25, 23.5, 22, and 21.5% for 3D-CRT, respectively (Fig. 6). IMRT gave the same results as those given above. For all target locations, the normal brain dose was significantly smaller in PBT plans compared to 3D-CRT and IMRT (both *p* = 0.000). There was no difference between IMRT and 3D-CRT (*p* = 0.649). There were significant positive correlations between target position and difference in normal brain dose in PBT compared to 3DCRT (*p* = 0.006) and IMRT (*p* = 0.004); if the target was more peripheral, there was a larger reduction in normal brain dose in PBT compared to radiotherapy.

## Discussion

Proton beam therapy reduces the low dose area for pediatric brain tumor, and it gradually becomes the common recognition [19]. Radiation induce emotional and behavioral deficits and those are remain long time [20]. There were reports indicate that the average dose to the brain is an important factor for future IQ [1]. In this study, we focused on the average dose to normal brain tissue, and we found that PBT can substantially reduce this dose, compared to IMRT and 3D-CRT. This result matches past reports [21, 22]. Table 2 shows the difference in the expected IQ after 10 years by using PBT instead of X-ray therapy. PBT was expected decreasing the degree of the 3.4 to 12.8 IQ point in ependymoma cases, and 1.3 to 5.3 point IQ point in germinoma cases. This advantage was found in local irradiation for ependymoma and whole-ventricle irradiation for germinoma, with reductions of almost 40 and 20%, respectively. This effect may produce a 5-point difference in IQ at entrance to senior high school for a patient with ependymoma who received radiotherapy at 4 years old, and a 3-point difference at high-school graduation for a patient with germinoma who received radiotherapy at age 13.

**Table 2:** Expected IQ difference after 10 years by using PBT instead of X-ray therapy

No.	Age (y)	Sex	Tumor	Prescribed dose (Gy)	Dose difference of the brain (%) (3D-CRT-PBT)	Dose difference of the brain (%) (IMRT-PBT)	Expected IQ difference (PBT-3DCRT)	Expected IQ difference (PBT-IMRT)
1.	6	M	Ependymoma	50.4	2.97	3.23	3.4	3.7
2.	6	M	Ependymoma	54.0	3.29	3.08	3.8	3.5
3.	2	F	Ependymoma	50.4	5.70	5.34	6.5	6.1
4.	3	F	Ependymoma	59.4	8.67	9.74	9.9	11.1
5.	4	M	Ependymoma	61.2	8.38	11.20	9.6	12.8
6.	3	F	Ependymoma	45.0	4.64	4.59	5.3	5.2
7.	11	M	Germinoma	30.6	3.92	3.34	4.5	3.8
8.	16	F	Germinoma	30.6	3.67	4.07	4.2	4.6
9.	10	M	Germinoma	30.6	3.73	4.28	4.3	4.9
10.	14	M	Germinoma	30.6	3.92	3.83	4.5	4.4
11.	14	F	Germinoma	24.0	2.88	1.34	3.3	1.5
12.	12	F	Germinoma	30.6	4.62	3.52	5.3	4.0

The advantages of PBT compared to photon radiotherapy including IMRT have been described in treatment of pediatric CNS tumors [9–11]. MacDonald et al. found that proton beams can achieve tumor coverage as well as IMRT, but that normal tissue sparing was better in PBT for patients with germ cell tumor and ependymoma [17, 18]. Our results are similar to these reports [5, 23]. In addition, PBT is more effective than IMRT or 3D-CRT for treatment of a large or peripheral tumor. For tumor location, the reduction in the dose to normal brain tissue was large when the target was outside over 2 cm from the brain center. Regarding the model case of the differential target position, we used sphere targets of diameters 4 cm, and this distance was equal to the target radius, which suggests that an advantage of PBT may emerge when the target does not cross the midline of the brain.

From the result of treated case and model case, we found no case that PBT has disadvantage compared with X-ray therapy about normal brain dose, but if the tumor was center and small, the benefit of using PBT was small relatively. Recently it was reported that age and mean radiation dose to specific brain volumes, including the temporal lobes and hippocampi, had a significant impact on longitudinal scores [24]. It is future problem to analyze the risk organ in vain, and it is necessary that assessing not only about brain tumors, but also about body tumors.

## Conclusion

PBT has maximum advantage about normal brain dose for a larger and peripheral tumor, and there was no case that PBT has disadvantage. The dose to normal brain tissue is lower with PBT compared with 3D-CRT and IMRT in local and whole-ventricle irradiation. This information matches past reports.
